# HTLV-1-associated uveitis mimicking thyroid-associated ophthalmopathy during antithyroid therapy: a diagnostic pitfall and management challenge

**DOI:** 10.1530/EDM-25-0177

**Published:** 2026-04-01

**Authors:** Daigo Kasamatsu, Etsuko Tanabe, Marie Okubo, Makiko Shimodaira, Yoshio Nakamura

**Affiliations:** Department of Diabetes and Endocrinology, Hyogo Prefectural Amagasaki General Medical Center, Amagasaki, Hyogo, Japan

**Keywords:** uveitis, HTLV-1, polyautoimmunity, Graves’ disease, thyroid-associated ophthalmopathy

## Abstract

**Summary:**

We report the case of a woman in her 70s, a known HTLV-1 carrier with recent-onset Graves’ disease, who developed HTLV-1-associated uveitis (HAU) four weeks after the initiation of methimazole therapy. The diagnosis was established based on characteristic ophthalmologic findings and exclusion of other causes. Her uveitis resolved completely following local steroid injection; however, subsequent management of Graves’ disease proved exceptionally challenging. Both methimazole and propylthiouracil eventually had to be discontinued because of safety concerns, including severe adverse reactions with propylthiouracil (granulocytopenia and hepatotoxicity), necessitating radioactive iodine therapy. During long-term follow-up, she developed Sjögren’s syndrome, illustrating the risk of polyautoimmunity in HTLV-1 carriers. This case highlights a diagnostic pitfall: in HTLV-1-endemic areas, new-onset visual disturbances during antithyroid drug treatment should not automatically be attributed to thyroid-associated ophthalmopathy, and HAU should be included in the differential diagnosis. It also underscores the need for careful monitoring for drug-related complications and the emergence of additional autoimmune disorders in this population.

**Learning points:**

- In HTLV-1 carriers, visual disturbances emerging shortly after the initiation of antithyroid drug therapy should not be automatically attributed to thyroid-associated ophthalmopathy; clinicians must differentiate HTLV-1-associated uveitis (HAU) to ensure appropriate management.
- Severe adverse reactions, including hepatotoxicity and granulocytopenia, can occur with antithyroid drugs, such as methimazole and propylthiouracil, and may arise sequentially, highlighting the significant therapeutic challenge and the necessity of careful monitoring in this population.
- HTLV-1 carriers diagnosed with one autoimmune disorder are at increased risk of developing polyautoimmunity, such as subsequent Sjögren’s syndrome, necessitating comprehensive and long-term multisystem surveillance.

## Background

HTLV-1 infection remains endemic in southwestern Japan, particularly in Kyushu, including Kagoshima. Kagoshima is recognized as one of the world’s highly endemic areas for HTLV-1, with a substantial carrier burden, especially among older individuals (1). HTLV-1-associated uveitis (HAU) is a recognized HTLV-1-associated inflammatory disorder and is among the common causes of uveitis in HTLV-1-endemic regions of Japan (2, 3). In a large clinical survey from southern Kyushu, an HTLV-1-endemic region that includes Kagoshima, HAU accounted for 17.1% of uveitis cases and was the most common defined etiology (4). Although a strong association between HAU and Graves’ disease has been reported, the specific triggers of uveitis remain unclear (5, 6, 7). Notably, uveitis may develop shortly after initiating antithyroid medication (5, 6, 8), raising the question of whether these treatments might act as triggers and, more importantly, creating a potential diagnostic pitfall. This case is particularly valuable because it details a complex clinical course involving HAU onset, multiple adverse drug reactions, and subsequent development of polyautoimmunity, providing essential educational insights for clinicians managing similarly challenging cases.

## Case presentation

A Japanese woman in her 70s from Kagoshima Prefecture – an area endemic for HTLV-1 – presented with acute visual disturbance in her left eye. Her medical history included recent-onset Graves’ disease, breast cancer, and known HTLV-1 carrier status, with a family history significant for HTLV-1-associated lymphoma. Four weeks before symptom onset, methimazole (15 mg/day) and potassium iodide (50 mg/day) treatment was initiated concurrently for Graves’ disease. At presentation, she described a one-week history of progressively worsening visual loss and floaters in the left eye.

## Investigation

**Figure 1:**
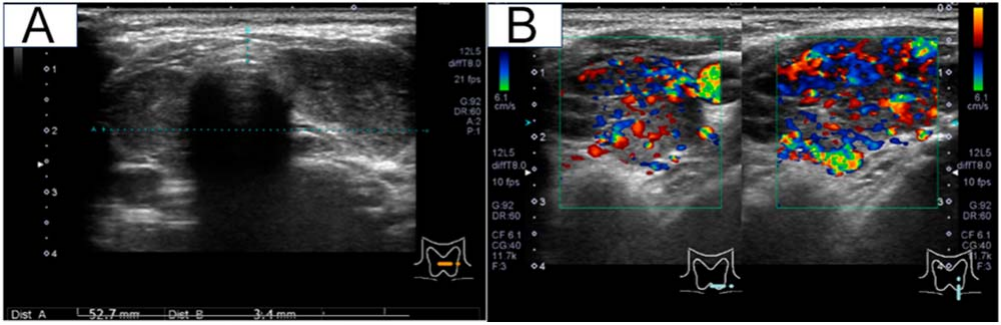
Ultrasonographic findings of the thyroid gland in the patient with Graves’ disease. (A) B-mode imaging shows diffuse goiter with a heterogeneous internal echotexture. (B) Color Doppler imaging reveals markedly increased parenchymal blood flow, a finding consistent with active Graves’ disease.

**Figure 2:**
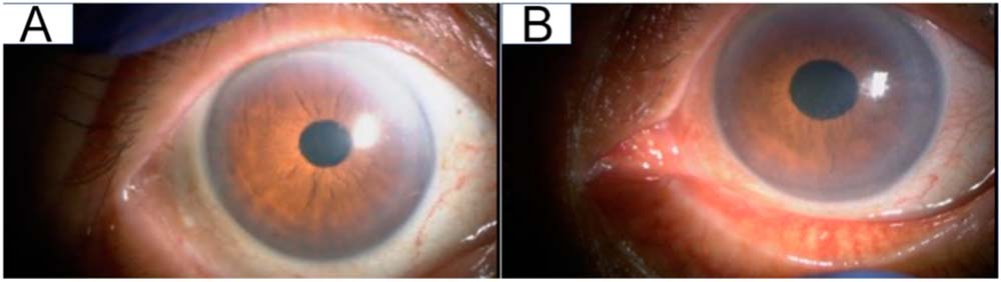
External ocular photographs at presentation. (A) Right eye: no obvious conjunctival injection or eyelid retraction is seen. (B) Left eye: conjunctival injection is present, without obvious eyelid retraction.

**Figure 3:**
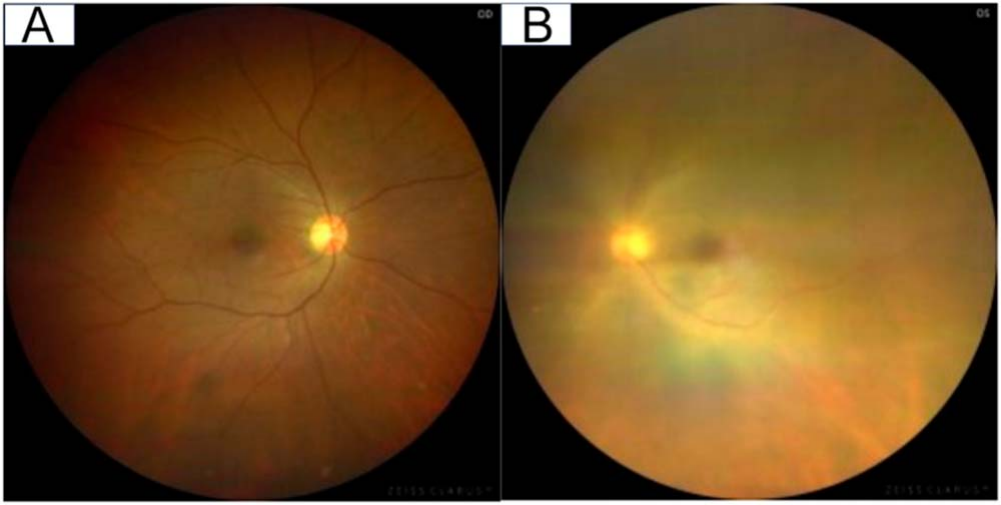
Comparison of fundus findings at presentation. (A) The right fundus is clear and unremarkable. (B) In contrast, the view of the left fundus is significantly obscured by dense vitreous opacities, consistent with active intraocular inflammation.

**Figure 4:**
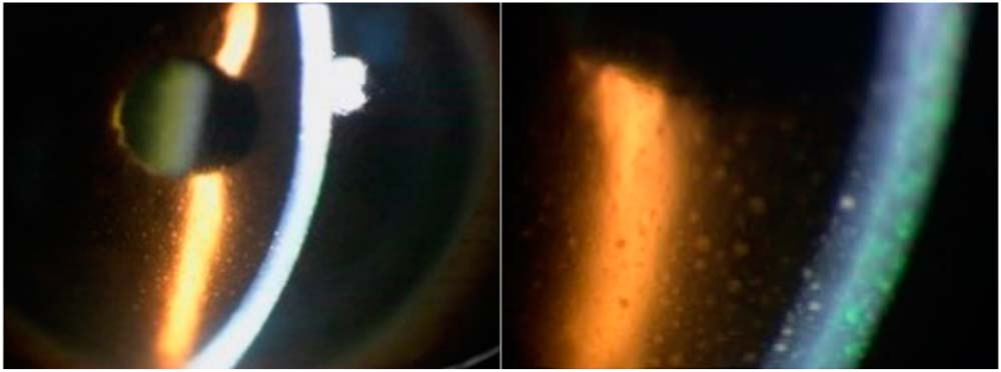
Slit-lamp microscopy of the left eye. The images reveal signs of anterior uveitis, including fine keratic precipitates deposited on the corneal endothelium and inflammatory cells floating in the anterior chamber.

Physical examination revealed a diffusely enlarged, non-tender thyroid gland (Fig. 1). There was no exophthalmos or limitation of extraocular movements. Orbital MRI was not performed; however, there were no clinical features of active thyroid-associated ophthalmopathy/thyroid eye disease, and the clinical activity score (CAS) was 0/7 in the right eye and 1/7 in the left eye (conjunctival injection) (Fig. 2). Ophthalmological evaluation demonstrated decreased best-corrected visual acuity in the left eye (0.2), compared to the unaffected right eye (0.9). Intraocular pressures were normal bilaterally (11.0 mmHg right; 12.7 mmHg left). Fundus examination of the left eye was markedly limited due to dense vitreous opacities (Fig. 3B), and slit-lamp examination showed fine keratic precipitates on the corneal endothelium and inflammatory cells within the anterior chamber (Fig. 4). Examination of the right eye was normal (Fig. 3A).

Initial laboratory evaluation at the diagnosis of Graves’ disease revealed severe hyperthyroidism: suppressed thyroid-stimulating hormone (TSH) < 0.01 µIU/mL, elevated free triiodothyronine (FT3) > 20.0 pg/mL, elevated free thyroxine (FT4) > 5.0 ng/dL, and high titers of TSH receptor antibody (TRAb: 26.7 IU/L) and thyroid-stimulating antibody (TSAb: 1,308%). Two weeks following initiation of methimazole therapy, thyroid function had significantly improved (TSH: < 0.01 µIU/mL; FT3: 4.29 pg/mL; FT4: 1.27 ng/dL), at which time no ocular symptoms were present. However, ocular symptoms appeared four weeks after starting methimazole, and at five weeks, thyroid hormone levels had further normalized (TSH:< 0.01 µIU/mL; FT3: 2.27 pg/mL; FT4: 0.71 ng/dL).

Subsequent investigations for uveitis revealed positive serum HTLV-1 antibody (123.5 S/CO by CLIA). Investigations for sarcoidosis – including serum angiotensin-converting enzyme (ACE) levels and chest x-ray – were negative or within normal limits. Given the clinical context, other potential causes of uveitis, such as Behçet’s disease, tuberculosis, and syphilis, were considered clinically unlikely; therefore, specific testing for these etiologies was not pursued. Based on these findings, intraocular inflammation was attributed to HTLV-1 infection, confirming a diagnosis of HAU. The diagnosis was based on HTLV-1 seropositivity, compatible ophthalmologic findings, and exclusion of other defined causes of uveitis, consistent with published diagnostic approaches (4, 5).

## Treatment

The patient received local steroid therapy (sub-Tenon injection of triamcinolone acetonide) for HAU, resulting in successful control of ocular inflammation. Management of Graves’ disease was more challenging. Given the close temporal association between antithyroid drug initiation and the onset of ocular inflammation, a drug-related contribution could not be completely excluded at the time of presentation. Therefore, methimazole was discontinued as a precautionary, safety-oriented decision while the differential diagnosis was being established. In retrospect, the subsequent clinical course supported HAU as the primary cause, and antithyroid drug discontinuation may not have been essential; however, the initial decision was considered reasonable in real-world practice. Antithyroid therapy was withheld for approximately four months after methimazole discontinuation, as thyroid hormone levels remained within acceptable ranges while carefully monitoring for adverse reactions. Subsequently, when laboratory evidence of recurrent thyrotoxicosis emerged, propylthiouracil (PTU) was initiated at the patient’s request. However, PTU led to liver injury (AST: 179 U/L, ALT: 344 U/L) as well as granulocytopenia (neutrophil count: 900/μL). Ultimately, radioactive iodine therapy was performed, successfully resolving her hyperthyroidism. A fixed activity of 13 mCi was selected according to institutional practice and guideline-consistent dosing (9). Given the high TRAb/TSAb titer and concern for post-RAI worsening of ophthalmopathy, prophylactic oral betamethasone (2 mg/day) was administered for 4 days starting on the day of radioiodine treatment. A short course was considered sufficient because the CAS was low (0/7 right, 1/7 left) and the risk of clinically significant post-RAI ophthalmopathy was judged to be limited.

## Outcome and follow-up

**Figure 5:**
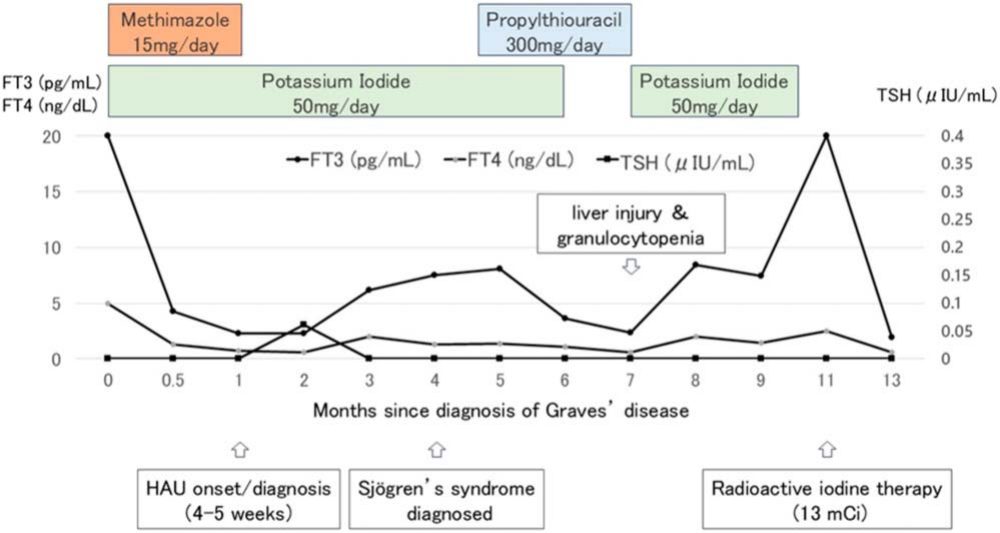
Clinical course of the patient. The graph illustrates the serial changes in serum TSH, free T3, and free T4 levels, plotted in months since the diagnosis of Graves’ disease (month 0). The bars at the top indicate the treatment periods for methimazole, potassium iodide (KI), and propylthiouracil (PTU). Key clinical events, including the onset of HAU, diagnosis of Sjögren’s syndrome, and major adverse events (liver injury and granulocytopenia, indicated by an arrow), are annotated along the timeline. FT3, free triiodothyronine; FT4, free thyroxine; HAU, HTLV-1-associated uveitis; KI, potassium iodide; PTU, propylthiouracil; TSH, thyroid-stimulating hormone.

The local steroid injection led to complete resolution of ocular inflammation and recovery of vision. At 36 months of follow-up, there has been no recurrence of HAU. However, she subsequently developed symptoms consistent with Sjögren’s syndrome, including dry mouth, ocular discomfort, and joint pain. Diagnostic tests confirmed elevated anti-SSA (44.2 U/mL) and anti-SSB (14.6 U/mL) antibodies, and significantly decreased salivary secretion (gum test: 1.8 mL in 10 min). Other autoimmune markers, including MPO-ANCA, PR3-ANCA, anti-dsDNA, anti-Sm, anti-U1RNP, anti-RNA polymerase antibodies, rheumatoid factor, and anti-CCP antibody, were negative. The overall clinical course is summarized in Fig. 5.

## Discussion

We present a notable case of HAU developing shortly after initiation of antithyroid therapy for Graves’ disease in an HTLV-1 carrier, complicated by subsequent polyautoimmunity and adverse drug reactions. Two significant clinical implications emerge from this case. Although the coexistence of HTLV-1 infection, Graves’ disease, and Sjögren’s syndrome is not novel in itself, this case uniquely underscores the complexities involved in managing sequential autoimmune disorders and adverse drug reactions in clinical practice.

First, the close temporal relationship between the initiation of methimazole and the onset of HAU raises important diagnostic considerations. While a strong association between Graves’ disease and HAU has been established previously (6, 7), the exact mechanism triggering ocular inflammation remains unclear. One hypothesis suggests that elevated thyroid hormone levels in Graves’ disease may increase HTLV-1 proviral load, thereby promoting viral gene expression (7). However, our patient developed uveitis as thyroid function normalized. Although some authors have speculated that antithyroid drugs might modulate immune responses (9, 10), in our case, a causal role of methimazole cannot be established, and the temporal association should mainly be regarded as a diagnostic pitfall. While previous reports have documented similar temporal associations (6, 8), direct causality remains speculative. Nonetheless, clinicians must maintain vigilance for HAU development during the early phase of antithyroid therapy, particularly in HTLV-1 endemic areas. A limitation of this report is that HTLV-1 proviral load measurement was not performed, which might have provided additional insights into disease severity and progression.

Second, our patient’s complex treatment course underscores the challenge of managing Graves’ disease in HTLV-1 carriers. Although PTU could theoretically offer an alternative to methimazole (9, 10), our patient experienced severe adverse reactions, including liver injury and granulocytopenia. Consequently, no definitive recommendation favoring PTU over methimazole can be made based on the current evidence (9, 10). Instead, this highlights the necessity for individualized treatment decisions and careful monitoring for potential drug reactions in these patients.

The subsequent development of Sjögren’s syndrome during follow-up further emphasizes the propensity of HTLV-1 carriers to develop polyautoimmunity. Previous studies have linked HTLV-1 infection to various autoimmune diseases, including Sjögren’s-like syndromes, suggesting broader immune dysregulation (5). Therefore, clinicians should implement long-term, comprehensive surveillance for autoimmune manifestations beyond initial disease management.

In conclusion, this case highlights critical educational points for clinicians. It underscores the importance of early suspicion and diagnosis of HAU in HTLV-1 carriers receiving antithyroid medications (6, 8), careful monitoring for adverse drug reactions (9, 10), and long-term vigilance for additional autoimmune disorders (5).

## Declaration of interest

The authors declare that there is no conflict of interest that could be perceived as prejudicing the impartiality of the research reported.

## Funding

This research did not receive any specific grant from any funding agency in the public, commercial, or not-for-profit sector.

## Patient consent

Written informed consent for publication of their clinical details and/or clinical images was obtained from the patient.

## Author contribution statement

DK was involved in the diagnosis and management of the patient and drafted the manuscript. YN critically reviewed and revised the manuscript. All authors reviewed and approved the final manuscript.
